# HBP1-mediated Regulation of p21 Protein through the Mdm2/p53 and TCF4/EZH2 Pathways and Its Impact on Cell Senescence and Tumorigenesis*[Fn FN2]

**DOI:** 10.1074/jbc.M116.714147

**Published:** 2016-04-21

**Authors:** Yifan Chen, Kewu Pan, Pingzhang Wang, Zhengyi Cao, Weibin Wang, Shuya Wang, Ningguang Hu, Junhui Xue, Hui Li, Wei Jiang, Gang Li, Xiaowei Zhang

**Affiliations:** From the ‡ Department of Biochemistry and Molecular Biology, School of Basic Medical Sciences, Beijing Key Laboratory of Protein Posttranslational Modifications and Cell Function, Peking University Health Science Center, Beijing 100191 and; the § Department of Immunology, School of Basic Medical Sciences, Peking University Health Science Center, Beijing 100191, China

**Keywords:** histone methylation, p53, senescence, ubiquitylation (ubiquitination), Wnt signaling, HBP1, p21, tumorigenesis

## Abstract

The activity of the CDK inhibitor p21 is associated with diverse biological activities, including cell proliferation, senescence, and tumorigenesis. However, the mechanisms governing transcription of p21 need to be extensively studied. In this study, we demonstrate that the high-mobility group box-containing protein 1 (HBP1) transcription factor is a novel activator of p21 that works as part of a complex mechanism during senescence and tumorigenesis. We found that HBP1 activates the p21 gene through enhancing p53 stability by inhibiting Mdm2-mediated ubiquitination of p53, a well known positive regulator of p21. HBP1 was also found to enhance p21 transcription by inhibiting Wnt/β-catenin signaling. We identified histone methyltransferase EZH2, the catalytic subunit of polycomb repressive complex 2, as a target of Wnt/β-catenin signaling. HBP1-mediated repression of EZH2 through Wnt/β-catenin signaling decreased the level of trimethylation of histone H3 at lysine 27 of overall and specific histone on the p21 promoter, resulting in p21 transactivation. Although intricate, the reciprocal partnership of HBP1 and p21 has exceptional importance. HBP1-mediated elevation of p21 through the Mdm2/p53 and TCF4/EZH2 pathways contributes to both cellular senescence and tumor inhibition. Together, our results suggest that the HBP1 transcription factor orchestrates a complex regulation of key genes during cellular senescence and tumorigenesis with an impact on protein ubiquitination and overall histone methylation state.

## Introduction

Cellular senescence is characterized by permanent cell cycle arrest and distinct morphological, physiological, and epigenetic changes in response to events such as telomere attrition, aberrant oncogene activation, and abrogation of tumor suppressor gene functions. Senescence is a tumor-suppressive process whose abrogation enables the path to tumorigenesis (1). Growing evidence suggests that senescence and tumorigenesis are crossed during tumor progression (2). A number of key senescence-associated factors, such as p53/p21 and RB/p16, have been found to prevent tumorigenesis (3–5). Furthermore, several distinct transcription factors that contribute to activating tumorigenesis, such as Zeb1, Twist, and Snail, can also concomitantly suppress senescence (6–8). Accordingly, it is important to directly regulate expression of these key factors. In addition to direct transcriptional regulation, epigenetic alterations also play an essential role in determining gene expression patterns and in setting the environment for activators or repressors to function appropriately. Histone methylation has been associated with cancer and senescence (9, 10).

The CDK^2^ inhibitor p21 is a critical factor in the inhibition of G_1_ progression and induction of senescence by blocking the activity of CDKs. Increased levels of p21 protein have been observed in contexts of cell cycle arrest, such as differentiation and senescence (11, 12). As one of the most well established p53 target genes, p21 is an essential mediator of p53-dependent cell cycle arrest. p21-depleted mouse embryonic fibroblasts are unable to undergo p53-dependent G_1_ arrest after DNA damage (13). The obvious dependence of p53 on p21 for the induction of cell cycle arrest and the established role of p21 as an inhibitor of proliferation suggest that p21 plays a crucial role in the induction of p53-dependent senescence and tumor inhibition. Indeed, lack of p21 abrogates senescence and induces transformation in several settings (14–16). The role of p21 is pivotal to the growth arrest of senescent and tumor cells; however, the mechanisms involved in regulating p21 expression remain incompletely understood. In this study, we found an unexpected connection to the HBP1 transcription factor, which our previous studies had linked to premature senescence and tumorigenesis.

HBP1 is a member of the sequence-specific high-mobility group (HMG) family, characterized by the DNA-binding domain known as HMG box (17, 18). By regulating the transcription of various genes, HBP1 has been found to participate in multiple cell progressions, including cell cycle inhibition, terminal differentiation, senescence induction, and tumor suppression, in a variety of tissues and cell types (19–21). In this study, we investigated the regulation of p21 by HBP1. We found that HBP1 enhanced p53 stability by inhibiting Mdm2-mediated ubiquitination of p53 and thus promoted p21 transcription activated by p53. On the other hand, HBP1 could also bind TCF4, an important transcription factor in Wnt/β-catenin signaling, through the repression domain and inhibit the transcriptional activation of the histone methyltransferase EZH2 by TCF4. EZH2 is the catalytic subunit of polycomb repressive complex 2 (PRC2), which catalyzes the methylation of histone H3 at lysine 27 (H3K27me) and mediates gene silencing of target genes via local chromatin reorganization (22). Overexpression of HBP1 reduced EZH2 levels and thus decreased the trimethylation of H3K27 on the p21 promoter, which further facilitated p53 binding on the p21 promoter and activated p21 transcription. Our study indicates that HBP1 potentially up-regulates p21 expression by both the Mdm2/p53 and TCF4/EZH2 pathways. The up-regulation of p21 by HBP1 induced cell growth arrest and cellular senescence and also inhibited tumorigenesis.

## Experimental Procedures

### 

#### 

##### Cell Culture, Transfection, siRNA and Lentivirus Gene Expression

The human cell lines H1299 (p53^−/−^), A549, HepG2, U2OS, HEK293T, and WI-38 were purchased from the ATCC and cultured in Dulbecco's modified Eagle's medium (Invitrogen) supplemented with 10% fetal bovine serum at 37 °C in 5% CO_2_. The GSK3β inhibitor SB415286 was purchased from Sigma, and LiCl was purchased from M&C Gene Technology. Cells were transiently transfected with plasmids using TurboFect transfection reagent (Thermo Scientific) following the protocol of the manufacturer. At 48 h after transfection, cells were harvested and lysed for detection of transfection efficiency. The siRNA was transfected with X-tremeGENE siRNA transfection reagent (Roche) following the protocol of the manufacturer. The β-catenin target siRNA sequence was 5-AGCTGATATTGATGGACAG-3. TCF4 target siRNA sequence was 5-AGACGAGGGCGAACAGGAG-3. The lentivirus vector pLL3.7-shHBP1 expresses shRNA that targets HBP1 mRNA (5′-ACTGTGAGTGCCACTTCTC-3′). The lentivirus vector pLL3.7-shp53 expresses shRNA that targets p53 mRNA (5′-CCACTTGAUGGAGAGTATT-3′). The lentivirus vector pLL3.7-EZH2 expresses shRNA that targets EZH2 mRNA (5′-GACTCTGAATGCAGTTGCT-3′).

##### Western Blotting and Antibodies

The experiment was performed as described previously (23). Cells were lysed in radioimmune precipitation assay buffer (Thermo Scientific) containing protease inhibitor mixture (Sigma), and protein concentrations were measured by the BCA protein assay kit (Pierce). A total of 20–60 μg of protein was separated by SDS-PAGE and transferred to nitrocellulose membranes (Pall). The primary antibodies used for immunoblotting analysis were against HBP1 (11746-1-AP, Proteintech), p53 (DO-1, sc-126, Santa Cruz Biotechnology; 9282, Cell Signaling Technology), Mdm2 (sc-965, Santa Cruz Biotechnology), p21 (K0081-3, MBL), GAPDH (KM9002, Sungene), EZH2 (5246, Cell Signaling Technology), Wnt2 (11160-1-AP, Proteintech), DNMT1 (sc-10222, Santa Cruz Biotechnology), p16 (10883-1-AP, Proteintech), FLAG (F1804, Sigma-Aldrich), HA (MMS-101P, Covance), GST (IT003M, M&C Gene Technology), His (66005-1, Proteintech), histone 3 (17168-1-AP, Proteintech), H3K27me3 (A2363, ABclonal), GFP(CW8006, CW Biotech), β-catenin (9587P, Cell Signaling Technology), TCF4 (13838-1-AP, Proteintech), and HDAC1 (AH379, Beyotime). The following secondary antibodies were purchased from Rockland: anti-mouse IgG antibody IRDye 800-conjugated (610-132-121) and DyLight 800-conjugated, affinity-purified anti-rabbit IgG (611-145-002). The infrared fluorescence image was obtained using an Odyssey infrared imaging system (LI-COR Bioscience, Lincoln, NE).

##### Real-time PCR

Total RNA was isolated using the RNAsimple total RNA kit (Tiangen). The cDNA was synthesized using the ReverAid first strand cDNA synthesis kit (Thermo Scientific) and then analyzed by real-time PCR analysis with Maxima SYBR Green qPCR Master Mix (Thermo Scientific). The DNA sequences of the primers were as follows: human HBP1, 5-TGAAGGCTGTGATAATGAGGAAGAT-3 and 5-CATAGAAAGGGTGGTCCAGCTTA-3 (these primers result in a 191-bp product); human p53, 5-TAACAGTTCCTGCATGGGCGGC-3 and 5-AGGACAGGCACAAACATGCACC-3 (these primers result in a 121-bp product); human p21, 5-GCAGACCAGCATGACAGATTT-3 and 5-GGATTAGGGCTTCCTCTTGGA-3 (these primers result in a 70-bp product); human Mdm2, 5-GCAGTGAATCTACAGGGACGC-3 and 5-ATCCTGATCCAACCAATCACC-3 (these primers result in an 83-bp product); human EZH2, 5-GGTTCAGACGAGCTGATGAAG-3 and 5-CGCTGTTTCCATTCTTGGTT-3 (these primers result in a 97-bp product); and human GAPDH, 5-CCATGGAGAAGGCTGGGG-3 and 5-CAAAGTTGTCATGGATGACC-3 (these primers result in a 195-bp product). GAPDH was applied as an internal control for normalizing the real-time PCR results.

##### Immunoprecipitation

Cells for the immunoprecipitation assay were lysed in IP lysis buffer (25 mm Tris-HCl (pH 7.4), 150 mm NaCl, 1% Nonidet P-40, 1 mm EDTA, and 5% glycerol) containing protease inhibitor mixture (Sigma). The cell extracts were incubated with the specified antibodies or control IgG and protein A-Sepharose (GE Healthcare) overnight at 4 °C with constant rotation. The immunocomplexes were then washed three times with IP lysis buffer, and subsequently resolved by SDS-PAGE followed by Western blotting analysis.

##### GST Pulldown Assay

The experiment was performed as described previously (23). The control GST and GST-tagged proteins were expressed in the *Escherichia coli* strain BL21 (DE3). The His-tagged recombinant protein expression vectors pET-HBP1, pET-Mdm2, and pET-p53, were constructed on the base of the pET-28b (+) vector. The vectors were transformed into BL21 (DE3) *E. coli*, and recombinant protein expression was induced by 0.1 mm isopropyl β-d-1-thiogalactopyranoside at 30 °C for 8 h. The purified control GST and GST-tagged proteins were incubated with glutathione-Sepharose beads (GE Healthcare) overnight at 4 °C, and then the His-tagged proteins were added to each tube for another 4 h at 4 °C. The beads were then washed three times with binding buffer and eluted with GST elution buffer. The elution was separated by SDS-PAGE, and the interactions were analyzed by Western blotting with the specified antibody.

##### In Vivo Ubiquitination Assay

For the detection of p53 ubiquitination levels, p53-null H1299 cells were cotransfected with various plasmids as indicated in individual experiments. 18 h after transfection, cells were treated with 10 μm MG132 (Merck Millipore) for 6 h, and the whole cell lysates prepared by FLAG lysis buffer (50 mm Tris-HCl (pH 7.8), 137 mm NaCl, 10 mm NaF, 1 mm EDTA, 1% Triton X-100, 0.2% Sarkosyl, 1 mm DTT, 10% glycerol, and fresh protease inhibitors) (24) were immunoprecipitated with anti-p53 antibody and then resolved by SDS-PAGE followed by Western blotting analysis.

##### Protein Half-life Assay

A549 cells were transfected with plasmids as indicated in individual experiments. 48 h after transfection, 100 μg/ml of cycloheximide was added to the dishes, and the cycloheximide treatment was terminated at the 0-, 20-, 40-, 60-, and 80-min time points as indicated. The whole cell lysates were prepared, and 25 or 60 μg of total protein from each sample was analyzed by Western blotting with anti-p53 antibody. Quantification of p53 protein was determined using TotalLab software normalized to GAPDH.

##### EMSA

The EMSA was conducted using the LightShift chemiluminescence EMSA kit (Thermo Scientific) following the instructions of the manufacturer. The nuclear extract (4 μg of protein) was mixed with a biotin-labeled DNA probe or cold competitor in binding buffer. The reaction mixture was incubated for 30 min at room temperature. Anti-FLAG antibody was included for supershifts. Then the reaction mixture was incubated for additional 30 min at room temperature. Samples were run on the 6% acrylamide gel at 70 V for 2 h. The gel was then transferred to a nylon membrane. The biotin-labeled DNA was detected using the streptavidin-horseradish peroxidase conjugate and LightShift chemiluminescent substrate. The DNA sequences of the probes used were as follows: EZH2-WT, 5-AGAGTTATATACTGCTTTGATTTGCT-3 and 5-AGCAAATCAAAGCAGTATATAACTCT-3; EZH2-MT, 5-AGAGTTATATACTGCGGGTATTTGCT-3 and 5-AGCAAATACCCGCAGTATATAACTCT-3.

##### ChIP

The ChIP assay was performed using a ChIP assay kit (Millipore) following the instructions of the manufacturer, using 2 μg of either normal IgG or antibodies against p53, HA, FLAG, and H3K27me3. Real-time PCR was performed as illustrated above at least three times with independent DNA samples. The reported data represent real-time PCR values normalized to input DNAs and to the values obtained with normal IgG, which were set as one unit in each calculation. Data are presented as -fold differences relative to input and values obtained by normal IgG with the formula 2^((Ct IgG − Ct Input) − (Ct Ab − Ct Input))^, where Ct is the threshold cycle, IgG is the normal IgG, Ab is the specific antibody, and Input is the input genomic DNA. For the TCF4 promoter, the PCR primer sequences were 5-CTAAACGTCTAGCAATTTAC-3 and 5-GCATTTTTAAGAGGAAGATC-3. For the p21 promoter, the PCR primer sequences were 5-CTGGACTGGGCACTCTTGTC-3 and 5-CTCCTACCATCCCCTTCCTC-3 (for p53 binding) (25) or 5-GTCTGCTGCA AATCTCAGTTTGCCC-3 and 5-GTGTGCAC GTAACAGAGCGCATCA-3 (for H3K27me3 binding) (26). For each antibody, at least three independent ChIP experiments were performed.

##### Reporter Gene Assay

Cells were transfected with plasmids as indicated in individual experiments. Cell lysates were prepared with the Dual-Luciferase reporter assay kit (Promega) according to the instructions of the manufacturer at 24–48 h post-transfection. The firefly luciferase activity measurements were normalized to *Renilla* luciferase activity for the same sample. The luciferase assay was performed on three biological replicates, and each replicate was measured at least three times.

##### Histone Extraction for Western Blotting

To identify histone modifications, acid extraction of histone was performed as reported previously (27). 24 h after transfection, H1299 cells were lysed in hypotonic lysis buffer (10 mm Tris-HCl (pH 8.0), 1 mm KCl, 1.5 mm MgCl_2_, and 1 mm DTT) containing protease inhibitor mixture (Sigma). The nuclei were then resuspended in 0.4 N H_2_SO_4_ and incubated for at least 30 min after spinning. The supernatant containing histones was collected and incubated with trichloroacetic acid on ice for 30 min. The histone pellet was collected after spinning, washed with acetone, and dissolved in diluted H_2_O.

##### MTT Assay

WI-38, A549, and p53-null H1299 cells were stably transfected with plasmids as indicated in individual experiment. After puromycin (0.4 μg/ml) and/or G418 (400 μg/ml) selection, cells were seeded into 96-well plates at a density of 2000 cells/well. After culturing for 1, 2, 3, 4, 5, 6, 7, 8, or 10 days, 15 μl of 3-(4,5-dimethylthyazol-2-yl)-2,5-diphenyltetrazolium bromide (MTT) solution (5 mg/ml) was added to each well, followed by further incubation at 37 °C for 4 h. The medium was removed and 200 μl of DMSO was added to each well to dissolve the formazan crystals. The absorbance at 490 nm was read using the microplate reader. The MTT assay was performed on three biological replicates, and each replicate was measured at least three times.

##### BrdU Incorporation in Situ

Cells were grown on coverslips and synchronized in 0.2% fetal bovine serum, Dulbecco's modified Eagle's medium for 24 h. The subconfluent cultures were incubated for 2 h in the presence of 10 μg of BrdU and fixed, and nuclei incorporating BrdU were visualized by immunostaining using a commercially available kit (BrdU labeling and detection kit, Roche). For visualization of all nuclei in a field, the coverslips were stained with Hoechst dye for 1 min at 37 °C. All coverslips were examined using fluorescence microscopy with the appropriate filters. At least 300 cells were counted in randomly chosen fields from each culture well.

##### Senescence-associated (SA) β-Gal Staining

The experiment was performed using a senescence β-galactosidase staining kit (Beyotime) following the instructions of the manufacturer. Cells were washed once in PBS, fixed for 15 min at room temperature in 3% formaldehyde, and washed three times with PBS again. Then, cells were incubated overnight at 37 °C with freshly prepared SA galactosidase stain solution. At least 300 cells were counted in randomly chosen fields (19).

##### Soft Agar Colony Formation Assay

The effect of HBP1 on the anchorage-independent growth of A549 and p53-null H1299 cells was estimated by a soft agar colony formation assay as described previously (23). Single-cell suspensions of 1.5–3 × 10^4^ cells were plated per 6-well plate in 2 ml of DMEM containing 10% FBS and 0.35% agar on a layer of 2 ml of the same medium containing 0.7% agar. Two weeks after culture, photographs were taken, and the numbers of colonies were determined by TotalLab software.

##### Tumorigenicity in Nude Mice

A549 and p53-null H1299 cells were stably transfected with either control plasmid or HBP1 plasmid or both HBP1 and EZH2 plasmid. After puromycin (0.4 μg/ml) and/or G418 (400 μg/ml) selection, 3 × 10^6^ cells were suspended in 150 μl of PBS and subcutaneously injected into the left or right hind leg of 6-week-old female nude mice. 3–4 weeks after injection, the mice were killed, the tumors were weighed, and the size was measured. Each cell subline was evaluated in three different animals.

##### Bioinformatics Analysis

All of the array data related to cancers and cancer lines from the Affymetrix human genome U133 Plus 2.0 platform were downloaded from the GEO database, and a TumorProfile database^3^ was developed to analyze the differentially expressed genes in tumors using data processing and microarray analysis methods described previously (28, 29). The expression profile of HBP1, EZH2, and p21 in a variety of cancers and the corresponding control (normal or non-tumor) tissues was searched in this database. Rank-based gene expression curves, which visually reflect the gene expression profile across multiple tissues, were generated using the TumorProfile dataset.

##### Statistical Analysis

The data are expressed as the mean ± S.E. from an appropriate number of experiments as indicated in the figure legends. The statistical analysis was done by using Student's *t* test, and *p* < 0.01 or 0.05 was considered significant.

## Results

### 

#### 

##### HBP1 Probably Promotes p21 Transcription by Regulating the Expression of p53 and EZH2

**FIGURE 1.**
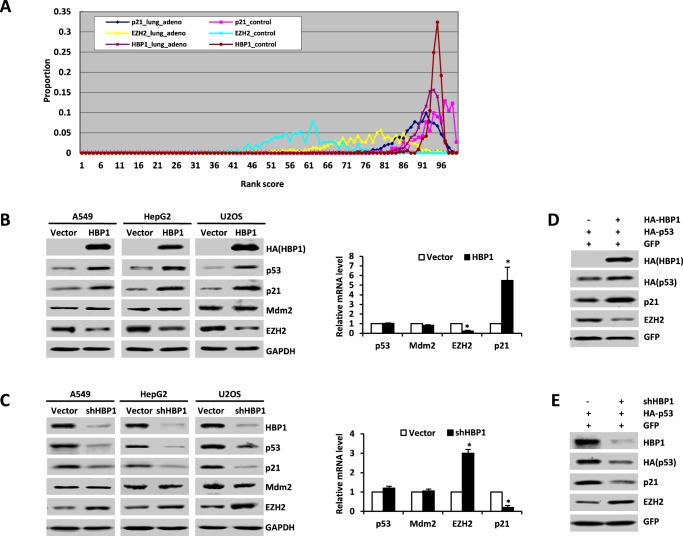
**Transcription factor HBP1 promotes p21 transcription, probably by regulating the expression of p53 and EZH2.** *A*, HBP1 and p21 are down-regulated at the mRNA level in lung adenocarcinoma according to bioinformatics analysis. In the rank-based gene expression curves, the *x* axis represents expressional intensity reflected by rank scores, whereas the *y* axis indicates sample percentiles at each rank score. *B*, HBP1 overexpression increases p53 and p21 expression and decreases EZH2 expression. The protein levels of HBP1, p53, p21, Mdm2, and EZH2 were measured by Western blotting in A549, HepG2, or U2OS cells stably transfected with PITA or PITA-HA-HBP1 through lentiviral infection. GAPDH was used as a loading control (*left panel*). The mRNA levels of p53, p21, Mdm2, and EZH2 were measured by real-time PCR in A549 cells stably transfected with PITA or PITA-HA-HBP1 through lentiviral infection. Results are representative of three independent experiments, and values are the mean ± S.E. *, *p* < 0.05. *C*, HBP1 knockdown by shRNA decreases p53 and p21 expression and increases EZH2 expression. The protein levels of HBP1, p53, p21, Mdm2, and EZH2 were measured by Western blotting in A549, HepG2, or U2OS cells stably transfected with pLL3.7-shHBP1 or pLL3.7 (as a control) through lentiviral infection. GAPDH was used as a loading control (*left panel*). The mRNA levels of p53, p21, Mdm2, and EZH2 were measured by real-time PCR in A549 cells transfected with pLL3.7-shHBP1 or pLL3.7. Results are representative of three independent experiments, and values are the mean ± S.E. *, *p* < 0.05. *D* and *E*, HBP1 enhances p53 protein expression at the posttranscriptional level. The protein levels of HBP1, p53, p21, and EZH2 were measured by Western blotting in H1299 cells cotransfected with HA-tagged p53 and GFP with or without HBP1 transfection (*D*). The protein levels of HBP1, p53, p21, and EZH2 were measured by Western blotting in H1299 cells cotransfected with HA-tagged p53 and GFP with or without HBP1shRNA transfection (*E*). The level of GFP is shown as equal transfection efficiency.

We had observed previously that p53 and p21 protein levels increased during HBP1-induced premature senescence and tumor inhibition in normal human fibroblasts (19–21), but the mechanism still has not been identified. Additionally, other work suggested that histone methyltransferase EZH2 depletion promotes cellular senescence by activation of p21 in gastric cancer cells (30). Intriguingly, we observed a statistically significant inverse correlation between HBP1 and EZH2 expression in cervical tumors from NCBI GEO database GSE9750 and in many cancer cell lines, such as HCT116 (a human colorectal cancer cell line) and A549 (a human lung carcinoma cell line), through data mining using the background dataset of the TumorProfile database (data not shown). In addition, we found that HBP1 and p21 were significantly down-regulated in lung adenocarcinoma, whereas EZH2 was significantly up-regulated in the cancer, according to bioinformatics analysis (Fig. 1*A*). Thus, we raised the question of whether p53 and EZH2 are both involved in p21 expression regulated by HBP1.

To determine whether HBP1 had a causative role in regulating p53, p21, and EZH2, we stably overexpressed HBP1 through lentiviral infection into three different cancer cell lines: A549 (lung cancer), HepG2 (liver cancer), and U2OS (osteosarcoma). As shown in Fig. 1*B*, exogenous HBP1 expression increased p21 protein and mRNA and increased p53 protein but had no effect on p53 mRNA. These results are consistent with a previous report showing that p53 is regulated primarily at the posttranscriptional level (31). Exogenous HBP1 also decreased EZH2 protein and mRNA levels in the three cell lines. To address the endogenous HBP1 and these genes, we used shRNA to knock down the HBP1 gene (Fig. 1*C*). HBP1 knockdown decreased p21 protein and mRNA, only decreased p53 protein, did not change p53 mRNA, and increased EZH2 protein and mRNA. Thus, HBP1 clearly regulates p21 and EZH2 at the transcriptional level and p53 at the posttranscriptional level.

We next asked whether the transactivation of p21 by HBP1 occurs through regulating the expression of p53 and EZH2. Because we reported previously that HBP1 inhibited tumor growth by up-regulating p16 expression (20), to prevent p16 from interfering, we chose lung cancer cell lines A549 (p16^−/−^) and H1299 (p16^−/−^ plus p53^−/−^) (32) as target cells to detect whether HBP1 had a causative role in promoting p21 expression. We expressed exogenous p53 in H1299 cells with HBP1 or HBP1 shRNA through lentiviral infection. As shown in Fig. 1, *D* and *E*, HBP1 increased exogenous p53 protein as well as the endogenous p21 protein and decreased endogenous EZH2 protein in H1299 cells (Fig. 1*D*), whereas HBP1 knockdown obtained the opposite result (Fig. 1*E*). In addition, EZH2 overexpression could attenuate HBP1-induced up-regulation of p21 (Fig. 5*D*), indicating that HBP1 probably also up-regulates p21 through inhibiting EZH2 expression. Taken together, these data suggest that HBP1 might promote p21 transcription by regulating the expression of p53 and EZH2.

##### HBP1 Promotes p21 Transcription by Enhancing p53 Protein Stability

**FIGURE 2.**
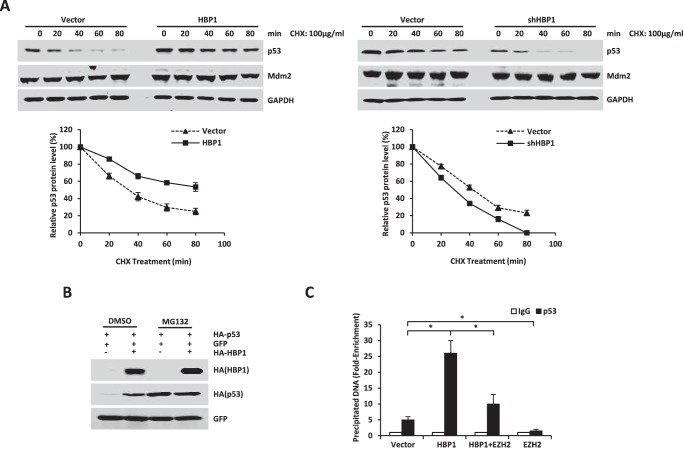
**HBP1 promotes p21 transcription by enhancing p53 protein stability.** *A*, HBP1 prolongs the half-life of p53. A549 cells were stably transfected with PITA, PITA-HA-HBP1 or pLL3.7, and pLL3.7-shHBP1 through lentiviral infection. Cells were incubated with the protein translation inhibitor cycloheximide (*CHX*) for 0, 20, 40, 60, or 80 min before harvest. P53 and Mdm2 protein levels were detected by Western blotting. GAPDH was used as the loading control for this turnover experiment (*top panel*). Densitometry is plotted for the average ± S.D. of three independent experiments (*bottom panel*). *B*, HBP1 does not elevate p53 levels in the presence of MG132. H1299 cells were cotransfected with p53 and GFP with or without HBP1. 42 h after transfection, cells were incubated with (*third* and *fourth lanes*) or without (*first* and *second lanes*) MG132 for another 6 h. p53 protein was detected by Western blotting. Level of GFP is shown as equal transfection efficiency. *C*, overexpression of HBP1 enhances the binding of p53 to the p21 promoter. H1299 cells were transfected with HA-tagged p53 and pcDNA3, pcDNA3-HA-HBP1, pcDNA3.1-His-EZH2, or both pcDNA3-HA-HBP1 and pcDNA3.1-His-EZH2. 24 h after transfection, cells were lysed for ChIP assay. The lysates were incubated with anti-p53 antibody or control IgG. The precipitated DNA fragments were amplified with specific oligonucleotides for the p21 promoter by real-time PCR. Results are representative of three independent experiments, and values are the mean ± S.E. *, *p* < 0.05.

Next, we explored the mechanism of how HBP1 up-regulates p53, a direct transactivator of p21. Because HBP1 regulates p53 expression at the posttranscriptional level, we investigated whether the increase in p53 protein by HBP1 was due to enhanced protein stability. As shown in Fig. 2*A*, overexpression of HBP1 caused a dramatic increase in p53 stability, whereas knockdown of HBP1 by shRNA caused an obvious reduction in p53 stability. Exogenous or endogenous HBP1 did not influence Mdm2 stability. Furthermore, HBP1 overexpression did not elevate p53 compared with the controlled cells in the presence of the proteasome inhibitor MG132 (Fig. 2*B*), which suggests that HBP1 up-regulates the p53 protein level in a proteasome-dependent manner.

To find out whether HBP1 also promotes p21 transcription activated by p53, we conducted a ChIP assay to detect the extent of p53 binding to the p21 promoter. As seen in Fig. 2*C*, HBP1 overexpression could enhance the binding of p53 onto the p21 promoter, which suggests that overexpression of HBP1 enhances p21 transactivation of p53. However, EZH2 overexpression could partially attenuate increased p53 binding to the p21 promoter caused by HBP1 overexpression. Hence, HBP1 might activate p21 transcription by promoting the binding of the p53 and p21 promoter.

##### HBP1 Stabilizes p53 by Inhibiting Mdm2-mediated p53 Ubiquitination and Degradation

We next sought to elucidate the potential mechanism by which HBP1 enhances p53 protein stability. The E3 ubiquitin ligase Mdm2 is the major negative regulator of the p53 level, so we first tested whether HBP1 up-regulates p53 by transcriptionally repressing Mdm2 expression. As shown in Fig. 1, *B* and *C*, and 2*A*, HBP1 overexpression and knockdown had no effect on either Mdm2 protein or mRNA levels. Although occasionally it could be seen that Mdm2 levels were slightly up-regulated by HBP1 expression (data not shown), this may be caused by p53 transcriptional activation. Thus, HBP1 does not up-regulate p53 by directly repressing Mdm2 expression or protein stability.

**FIGURE 3.**
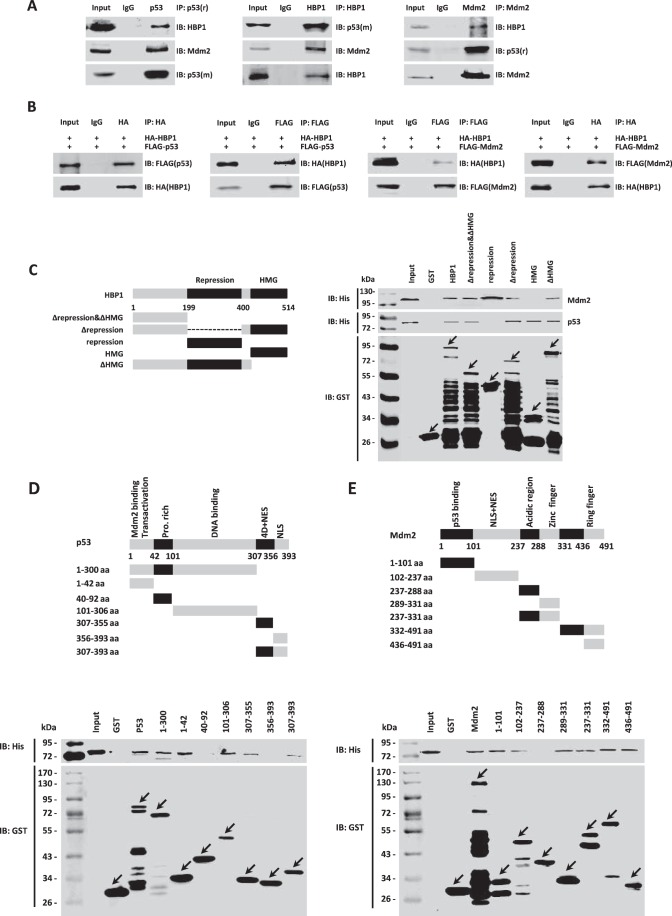
**HBP1 interacts with both p53 and Mdm2 *in vivo* and *in vitro*.** *A* and *B*, HBP1 interacts with both p53 and Mdm2 *in vivo*. A549 cells were lysed with IP lysis buffer and then subjected to immunoprecipitation with anti-p53 (9282), HBP1, or Mdm2 antibodies followed by Western blotting analysis (*A*). H1299 cells were cotransfected with HA-HBP1 and FLAG-p53 or FLAG-Mdm2. The IP assay was carried out by using anti-HA/FLAG antibody and followed by Western blotting with anti-FLAG/HA antibody. The same samples were immunoblotted (*IB*) against HA/FLAG to determine immunoprecipitation efficiency (*B*). *C–E*, HBP1 interacts with both p53 and Mdm2 *in vitro*. Shown is a schematic of N-terminal GST-tagged, full-length HBP1 along with its various deletion mutants (*C*, *left panel*), p53 along with its various deletion mutants (*D*, *top panel*), and Mdm2 along with its various deletion mutants (*E*, *top panel*). A GST pulldown assay was carried out to determine the domain of HBP1 essential for its interaction with p53 and Mdm2 (*C*, *right panel*), the domain of p53 essential for its interaction with HBP1 (*D*, *bottom panel*), and the domain of Mdm2 essential for its interaction with HBP1 (*E*, *bottom panel*). GST pulldown efficiency was evaluated by Western blotting with anti-GST antibody.

Because HBP1 and p53 both function as transcription factors in the nucleus, and Mdm2-mediated p53 ubiquitination also occurs early in the nucleus (33), we assumed that there may be physical interactions among the HBP1, p53, and Mdm2 proteins. First, we carried out the co-immunoprecipitation experiment with A549 cells. Cells were harvested and lysed in IP lysis buffer, and the whole cell lysates were subjected to immunoprecipitation with anti-p53, HBP1, or Mdm2 antibodies, followed by Western blotting analysis. As shown in Fig. 3*A*, the endogenous HBP1 interacted with both endogenous p53 and Mdm2 in A549 cells. Then we cotransfected HA-tagged HBP1 and FLAG-tagged p53 or FLAG-tagged Mdm2 into p53-null H1299 cells. 24 h after transfection, cells were harvested and lysed in IP lysis buffer. Then the cell lysates were immunoprecipitated with antibodies against HA or FLAG, followed by Western blotting analysis. As shown in Fig. 3*B*, the exogenous HBP1 also interacted with both exogenous p53 and Mdm2 in H1299 cells.

To determine whether the interaction between HBP1 and p53 or Mdm2 is a direct interaction, we conducted a GST pulldown assay with purified His-HBP1, His-p53, His-Mdm2, GST-HBP1, GST-p53, GST-Mdm2 proteins. As indicated in Fig. 3, *C–E*, the Western blotting results indicated that GST-p53 and GST-Mdm2, but not GST alone, pulled down HBP1 *in vitro*. The following reciprocal GST pulldown assay further testified to the interaction between HBP1 and p53 or Mdm2 *in vitro*. In summary, it can be concluded that HBP1 directly interacts with both p53 and Mdm2 *in vivo* and *in vitro*.

To further determine the specific domain required for their interaction, a set of deletion mutants of HBP1, p53, and Mdm2 were constructed in the pGEX-4T-1 vector, followed by GST pulldown assay as described above. As shown in Fig. 3*C*, the repression domain of HBP1 interacted with Mdm2 but not with p53. The following reciprocal GST pulldown assay showed that the two mutants 40–92 and 356–393 of p53 did not bind HBP1, whereas the other p53 mutants all bound HBP1 (Fig. 3*D*). For Mdm2, only mutant 237–288 did not bind HBP1 protein, whereas the other mutants all bound HBP1 (Fig. 3*E*). These results suggest that the interaction between HBP1 and p53 can be mediated by the Mdm2-binding domain, DNA binding domain, and the 4D domain of p53 but not the repression domain of HBP1. Also, the interaction between HBP1 and Mdm2 protein can be mediated by the p53-binding domain, acidic domain, and zinc finger domain of Mdm2 and the repression domain of HBP1.

**FIGURE 4.**
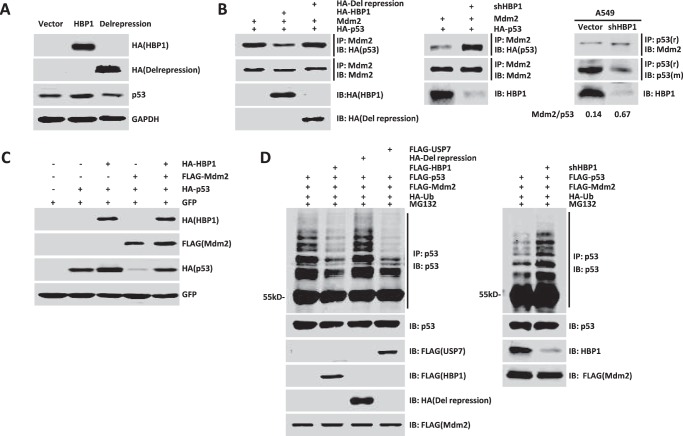
**HBP1 inhibits Mdm2-mediated p53 ubiquitination and degradation.** *A*, HBP1 up-regulates p53 through the repression domain. A549 cells were transfected with pcDNA3, pcDNA3-HA-HBP1, or pcDNA3-HA-Delrepression. P53 protein was detected by Western blotting. *B*, HBP1 inhibits the interaction between p53 and Mdm2 through the repression domain. H1299 cells were transfected with p53 + Mdm2 + pcDNA3, p53 + Mdm2 + HBP1, or p53 + Mdm2 + Delrepressin. The IP assay was carried out by using anti-Mdm2 antibody followed by Western blotting with anti-HA antibody (*left panel*). HBP1 knockdown by shRNA enhances interaction between p53 and Mdm2. H1299 cells were transfected with p53 + Mdm2 + pLL3.7 or p53 + Mdm2 + shHBP1. The IP assay was carried out by using anti-Mdm2 antibody followed by Western blotting with anti-HA antibody (*center panel*). A549 cells were transfected with pLL3.7 or shHBP1, and the IP assay was carried out by using anti-p53 antibody (9282) followed by Western blotting with anti-Mdm2 and anti-p53 (DO-1) antibodies. Quantification of p53 and Mdm2 protein was determined using TotalLab software (*right panel*). *IB*, immunoblot. *C*, Mdm2 inhibits HBP1-induced p53 up-regulation. Levels of p53 and GFP (as a control) were detected by Western blotting in H1299 cells (containing exogenous p53 expression) cotransfected with pcDNA3, HBP1, Mdm2, or HBP1 and Mdm2. *D*, HBP1 inhibits Mdm2-mediated p53 ubiquitination through the repression domain. H1299 cells were cotransfected with control vector, HBP1, Delrepression or USP7 (as a positive control) with FLAG-Mdm2, HA-ubiquitin, and HA-p53 for 18 h and then exposed to 10 μm MG132 for another 6 h prior to lysis. p53 protein was then isolated by immunoprecipitation and analyzed by anti-p53 antibody. HBP1 knockdown by shRNA enhances Mdm2-mediated p53 ubiquitination. H1299 cells were cotransfected with control vector or shHBP1 with FLAG-Mdm2, HA-ubiquitin, and HA-p53 for 18 h and then exposed to 10 μm MG132 for another 6 h prior to lysis. p53 protein was then isolated by immunoprecipitation and analyzed by anti-p53 antibody.

It has been found that HBP1 inhibits TCF4 transcriptional activation by interacting with TCF4 through its repression domain (34), and because HBP1 interacts with the p53 binding domain of Mdm2, we assumed that HBP1 also inhibited the interaction of Mdm2 and p53 by its repression domain. We first investigated whether the repression domain is of key importance in HBP1 up-regulating the p53 level. We overexpressed HA-tagged wild-type HBP1 and repression domain-deleted HBP1 (Delrepression) in A549 cells. As shown in Fig. 4*A*, the wild-type HBP1 elevated the p53 protein level, whereas Delrepression did not change the p53 protein level. Therefore, the repression domain of HBP1 is crucial for its enhancement on p53 expression.

We then cotransfected Mdm2 and HA-tagged p53 into H1299 cells with overexpression or knockdown of HBP1. 24 h after transfection, cells were harvested and lysed in IP lysis buffer. Then the lysates were immunoprecipitated with anti-Mdm2 antibody, and p53 that bound to Mdm2 was detected by Western blotting with anti-HA antibody. As shown in Fig. 4*B*, overexpression of HBP1 disrupted the interaction between exogenous p53 and Mdm2 (*left panel*), whereas knockdown of HBP1 enhanced exogenous p53 interaction with Mdm2 (*center panel*). However, Delrepression had no effect on the interaction between p53 and Mdm2 (Fig. 4*B*, *left panel*). We then reinforced this conclusion with A549 cells in which HBP1 was knocked down by shRNA and conducted the co-immunoprecipitation assay with anti-p53 antibody. As shown in Fig. 4*B*, knockdown of HBP1 also enhanced endogenous p53 interaction with Mdm2 in A549 cells (*right panel*). Therefore, HBP1 disrupts the interaction of p53 and Mdm2 through its repression domain. In addition, it was reported that Mdm2 could inhibit p53 transactivation function by binding to the transactivation domain of p53 (35, 36). Hence, disruption of p53/Mdm2 interaction by HBP1 might also release the p53 transcriptional activity inhibited by Mdm2 and thus promote p21 transcription.

Next we sought to determine whether HBP1 inhibits Mdm2-mediated p53 ubiquitination. We cotransfected HBP1, Mdm2, or both HBP1 and Mdm2 with p53 into H1299 cells. As shown in Fig. 4*C*, Mdm2 alone decreased the p53 protein level, whereas HBP1 partially rescued the p53 level decreased by Mdm2 in the absence of MG132. We then introduced exogenous Mdm2, p53, and HBP1 into H1299 cells. Cells were harvested 24 h after transfection before treatment with 10 μm proteasome inhibitor MG132 for 6 h, and ubiquitinated p53 was detected by Western blotting assay. As shown in Fig. 4*D*, Mdm2 ubiquitinated p53, HBP1 expression inhibited p53 ubiquitination, and the Delrepression mutant had no effect on p53 ubiquitination, with USP7 expression as a positive control (*left panel*). USP7 can lead to the deubiquitination and stabilization of p53 by inhibiting Mdm2 (37). Accordingly, p53 ubiquitination was increased in cells with HBP1 knockdown by shRNA (Fig. 4*D*, *right panel*). Together, these results suggested that HBP1 enhances p53 stability by inhibiting Mdm2-mediated p53 ubiquitination and degradation through the repression domain.

##### HBP1 Also Enhances p21 Transcription by Inhibiting EZH2 Expression in the Absence of p53

**FIGURE 5.**
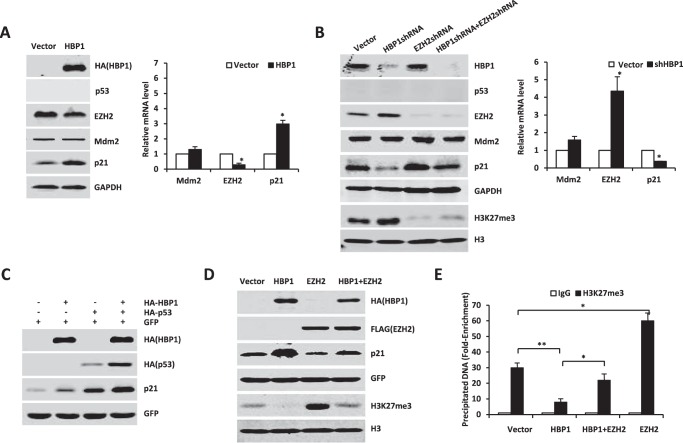
**HBP1 also enhances p21 transcription in the absence of p53.** *A–C*, HBP1 regulates p21 and EZH2 expression in the absence of p53. *A*, H1299 cells were transfected with pcDNA3 or pcDNA3-HA-HBP1. The protein levels of p21, Mdm2, and EZH2 were detected by Western blotting (*left panel*), and mRNA from the same cells was extracted and analyzed by real-time PCR. Results are representative of three independent experiments, and values are the mean ± S.E. *, *p* < 0.05. *B*, H1299 cells were transfected with pLL3.7, pLL3.7-shHBP1, pLL3.7-shEZH2, or pLL3.7-shHBP1 + pLL3.7-shEZH2. The protein levels of p21, Mdm2, EZH2, and H3K27me3 were detected by Western blotting (*left panel*), and mRNA from the same cells was extracted and analyzed by real-time PCR. Results are representative of three independent experiments, and values are the mean ± S.E. *, *p* < 0.05. *C*, H1299 cells were transfected with pcDNA3 or pcDNA3-HA-HBP1 with (*third* and *fourth lanes*) or without (*second lane*) HA-p53. p21 protein was detected by Western blotting. *D*, EZH2 rescues the p21 protein elevation and overall H3K27me3 decrease caused by HBP1. H1299 cells were transfected with control vector, HBP1, EZH2, or HBP1 + EZH2. The protein levels of p21 and H3K27me3 were detected by Western blotting. *E*, HBP1 decreases the level of H3K27me3 on the p21 promoter. H1299 cells were transfected with control vector, HBP1, EZH2, or HBP1 + EZH2. 24 h after transfection, we conducted a ChIP assay with anti-H3K27me3 antibody or control IgG. The precipitated DNA fragments were amplified with specific oligonucleotides for the p21 promoter by real-time PCR. Results are representative of three independent experiments, and values are the mean ± S.E. *, *p* < 0.05; **, *p* < 0.01.

The results above together indicated that HBP1 up-regulated p21 through the p53/Mdm2 pathway. Interestingly, we also found that HBP1 is able to elevate p21 protein and mRNA levels in the absence of p53 (Fig. 5*A*). Furthermore, HBP1 could also enhance the exogenous p53-induced elevated p21 level (Fig. 5*C*). The knockdown of HBP1 decreased p21 protein and mRNA levels in the absence of p53 (Fig. 5*B*). All of these data suggest that there might be at least one other pathway participating in the regulation of p21 by HBP1 and that this pathway could enhance the up-regulation of p21 by p53. According to our data, HBP1 also repressed EZH2 expression, and EZH2 overexpression could attenuate HBP1-induced up-regulation of p21 (Fig. 5*D*), which indicates that HBP1 up-regulates p21 partly through inhibiting EZH2 expression. In fact, accumulating evidence indicates that histone methylation also regulates gene transcription via local chromatin reorganization, in which the histone methyltransferase EZH2 plays an important part (38, 39). Hence, we hypothesized that HBP1 might also up-regulate the p21 level through inhibiting EZH2.

As indicated in Figs. 1, *B* and *C*, and 5, *A* and *B*, overexpression of HBP1 decreased both EZH2 protein and mRNA levels, whereas knockdown of HBP1 elevated EZH2 protein and mRNA levels, which were observed both in the presence and absence of p53. Thus, HBP1 inhibited EZH2 expression independently of p53 at the transcriptional level. However, in p53-null H1299 cells, overexpression of EZH2 caused a decrease in the level of p21 protein (Fig. 5*D*, compare the *third lane* with the *first lane*) and partially rescued the increase of p21 caused by HBP1 (Fig. 5*D*, compare the *fourth lane* with the *second lane*). Hence, HBP1 could also up-regulate the level of p21 by down-regulating EZH2 expression in a p53-independent manner. To determine the molecular mechanism by which HBP1 controls p21 through the EZH2 pathway, p53-null H1299 cells were transfected with HBP1, EZH2, or both HBP1 and EZH2. 24 h after transfection, cells were harvested, and the histones were extracted to detect the trimethylation of histone H3. As shown in Fig. 5*D*, overexpression of HBP1 decreased the overall level of H3K27me3, whereas EZH2 increased the overall level of H3K27me3 and partially rescued the decrease of H3K27me3 caused by HBP1. Knockdown of HBP1 increased the overall level of H3K27me3 (Fig. 5*B*). However, when EZH2 was also knocked down, the decrease of p21 and increase of H3K27me3 caused by HBP1 knockdown were partially rescued (Fig. 5*B*, compare the *fourth lane 4* with the *second lane*). In addition, EZH2 knockdown also attenuated the increases in cell growth and colony formation in soft agar caused by HBP1 knockdown, as assessed by MTT assay and soft agar assay (supplemental Fig. S1). Our data indicated that depletion of EZH2 could rescue the protein changes of p21 and H3K27me3 caused by HBP1 knockdown, thus rescuing the phenotype in HBP1-depleted cells. Then we performed a ChIP assay to detect the effect of HBP1 on the level of H3K27me3 on the p21 promoter. As shown in Fig. 5*E*, overexpression of HBP1 also decreased the level of H3K27me3 on the p21 promoter. These results suggest that HBP1 decreases the level of H3K27me3 on the p21 promoter by inhibiting EZH2 expression, thus activating p21 transcription.

##### The EZH2 Gene Is a Novel Target of Wnt/β-Catenin Signaling

**FIGURE 6.**
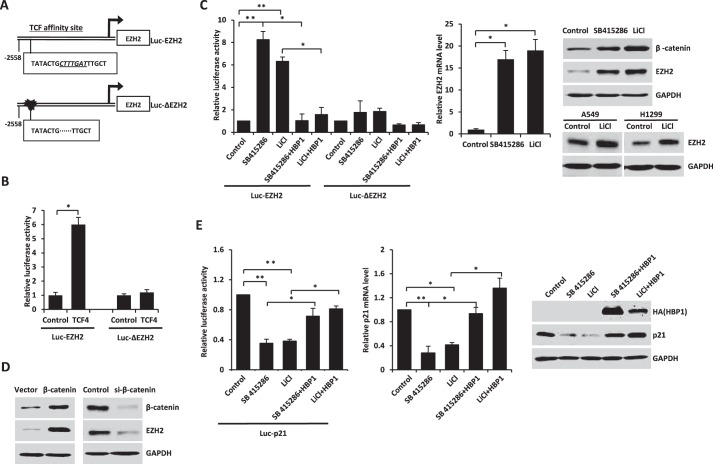
**The EZH2 gene is a novel target of Wnt/β-catenin signaling.** *A*, schematic of the EZH2 promoter. Shown is the LEF/TCF affinity site within the EZH2 promoter at positions −2533 to −2551 from the transcriptional start site (*Luc-EZH2*, *top panel*). Luc-ΔEZH2 is a mutant EZH2 promoter with a deletion in the LEF/TCF affinity site (*bottom panel*). *B*, the integrity of the affinity site is indispensable for TCF4 to enhance the EZH2 promoter. HEK293T cells were cotransfected with 0.1 μg of the indicated reporters and 0.6 μg of TCF4 expression plasmids. The luciferase activities were expressed as mean ± S.E. from four experiments. *, *p* < 0.05. *C*, the activation of Wnt/β-catenin signaling increases EZH2 expression. The integrity of the affinity site is indispensable for Wnt/β-catenin signaling activators enhancing the EZH2 promoter. HEK293T cells transfected with Luc-EZH2 or Luc-ΔEZH2 were treated with Wnt/β-catenin signaling activators of SB415286 or LiCl with or without HBP1. The luciferase activities were expressed as mean ± S.E. from four experiments (*left panel*). *, *p* < 0.05; **, *p* < 0.01. HEK293T cells were treated with SB415286 or LiCl. Real-time PCR was performed to detect the EZH2 mRNA level (*center panel*). Results are representative of three independent experiments, and values are the mean ± S.E. *, *p* < 0.05. Western blotting was performed to detect β-catenin and EZH2 protein levels (*right panel*, *top*). A549 and H1299 cells were treated with LiCl, and Western blotting was performed to detect EZH2 protein levels (*right panel*, *bottom*). *D*, exogenous or endogenous β-catenin regulates EZH2 expression. The protein levels of β-catenin and EZH2 were measured by immunoblotting in HEK293T cells transfected with β-catenin expression plasmid or β-catenin siRNA. *E*, the activation of Wnt/β-catenin signaling decreases p21 expression, which is rescued by HBP1 overexpression. HEK293T cells were cotransfected with Luc-p21 and pcDNA3 or pcDNA3-HA-HBP1, and 24 h after transcription, the cells were treated with or without SB415286 or LiCl for another 24 h. The luciferase activities were expressed as mean ± S.E. from four experiments. *, *p* < 0.05; **, *p* < 0.01 (*left panel*). H1299 cells were transfected with pcDNA3 or pcDNA3-HA-HBP1, and 24 h after transcription, the cells were treated with or without SB415286 or LiCl for another 24 h. Real-time PCR was performed to detect the p21 mRNA level. Results are representative of three independent experiments, and values are the mean ± S.E. *, *p* < 0.05; **, *p* < 0.01 (*center panel*). Western blotting was performed to detect p21 protein level (*right panel*).

We next sought to find out the mechanism of EZH2 inhibition by HBP1. Using bioinformatic methods, we found a high-affinity site of TCF4, a transcription factor in Wnt/β-catenin signaling, on the EZH2 promoter (Fig. 6*A*). Thus we hypothesized that TCF4 might be a transcriptional activator for EZH2 gene.

Using Transfac, we found a potential TCF4 binding site (CTTTGAT) at positions −2538 to −2544bp from the transcriptional start site on the EZH2 promoter. We designed two EZH2 promoter-luciferase reporters with either a native EZH2 segment (2608 bp, from +50 bp to −2558 bp, Luc-EZH2) or with a deletion that abolishes the TCF4 affinity site (2601 bp, from +50 bp to −2558 bp, Luc-ΔEZH2) (Fig. 6*A*). HEK293T cells were cotransfected with TCF4 and Luc-EZH2 or Luc-ΔEZH2. As shown in Fig. 6*B*, TCF4 expression activated the EZH2 promoter (Luc-EZH2), whereas there was no effect on the EZH2 promoter that lacked the TCF4 affinity site by deletion (Luc-ΔEZH2). Thus, TCF4 activated EZH2 expression through the TCF4 high-affinity site on EZH2 promoter.

Because TCF4 is an important transcription factor in Wnt/β-catenin signaling (34), we next asked whether EZH2 is a novel target of Wnt/β-catenin signaling through TCF4 binding. LiCl and SB415286 are specific inhibitors of GSK3β, which can inhibit Wnt/β-catenin signaling. Therefore, LiCl and SB415286 can specifically activate Wnt/β-catenin signaling (40). We transfected HEK293T cells with Luc-EZH2 or Luc-ΔEZH2, and, 24 h after transfection, we incubated the transfected HEK293T cells with LiCl or SB415286 for another 24 h. Then the cells were lysed for the luciferase assay. As indicated in Fig. 6*C*, both LiCl and SB415286 activated the wild-type Luc-EZH2 but had no effect on the Luc-ΔEZH2 (*left panel*). Accordingly, EZH2 protein and mRNA levels were increased by LiCl and SB415286 (Fig. 6*C*, *center* and *right panels*). In the meantime, β-catenin, the downstream target of GSK3β in the Wnt/β-catenin signaling pathway, was also elevated by LiCl and SB415286 (Fig. 6*C*, *right panel*). Overexpression or knockdown of β-catenin led to the increase or decrease of EZH2 protein, respectively (Fig. 6*D*). In summary, these results identify that EZH2, as a novel target of TCF4, is involved in Wnt/β-catenin signaling.

Then we detected whether activated Wnt/β-catenin signaling could also lead to the decrease of p21 transcription. As shown in Fig. 6*E*, the p21 promoter was inhibited by LiCl and SB415286 (*left panel*), and accordingly, p21 mRNA and protein levels were decreased by LiCl and SB415286 (*center* and *right panels*). Thus, the activation of Wnt/β-catenin signaling leads to the decrease of p21 expression.

##### HBP1 Decreases EZH2 Expression by Inhibiting Wnt/β-Catenin Signaling

It has been known that HBP1 is a suppressor of Wnt/β-catenin signaling by inhibiting LEF/TCF binding to the target genes (34), hence we next sought to determine whether HBP1 inhibits EZH2 by blocking Wnt/β-catenin signaling.

**FIGURE 7.**
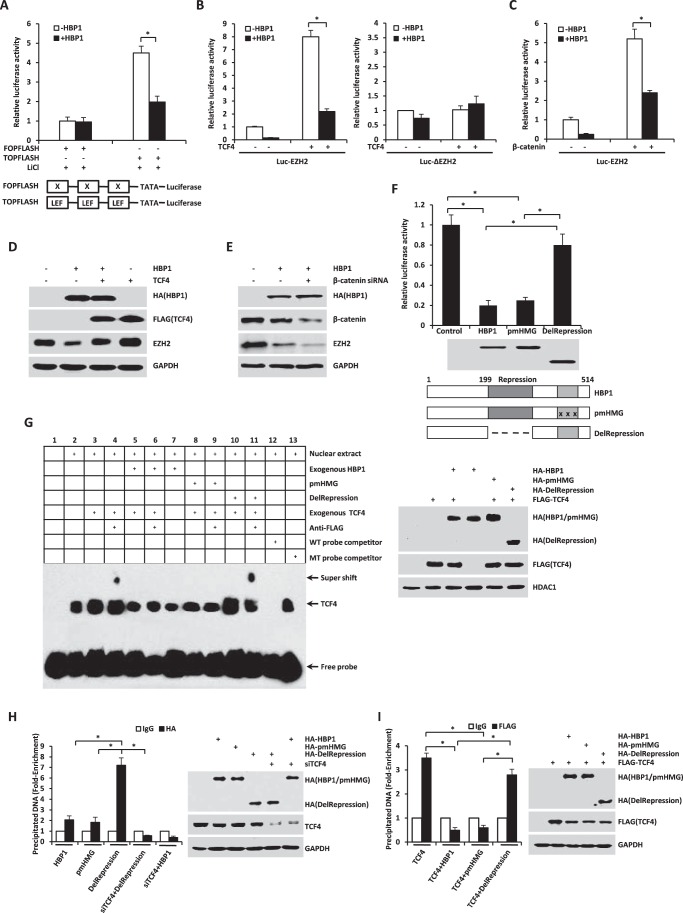
**HBP1 decreases EZH2 expression by inhibiting Wnt/β-catenin signaling.** *A*, HBP1 inhibits LiCl-activated gene expression through the LEF/TCF DNA binding site. HEK293T cells transfected with FOPFLASH or TOPFLASH were treated with LiCl for 12 h. Cells were harvested for the luciferase assay. TOPFLASH contains three LEF/TCF sites followed by the minimal TATA and luciferase. FOPFLASH contains three mutated LEF/TCF sites followed by the minimal TATA and luciferase. The luciferase activities were expressed as mean ± S.E. from four experiments. *, *p* < 0.05. *B* and *C*, HBP1 inhibits TCF4 or the β-catenin-activated EZH2 promoter. HEK293T cells were cotransfected with Luc-EZH2 or Luc-ΔEZH2 and TCF4 (*B*) or the β-catenin (*C*) expression plasmid. The luciferase activities were expressed as mean ± S.E. from four experiments. *, *p* < 0.05. *D*, TCF4 rescues HBP1 inhibition of EZH2 expression. Levels of EZH2 and GAPDH (as a control) were determined by Western blotting in HEK293T cells transfected with HBP1, TCF4, or HBP1 and TCF4. *E*, β-catenin knockdown by siRNA enhances HBP1 inhibition of EZH2 expression. Levels of β-catenin, EZH2, and GAPDH (as a control) were determined by Western blotting in HEK293T cells transfected with HBP1 with or without β-catenin siRNA. *F*, repression domain is indispensible for HBP1 inhibition of the EZH2 promoter. HEK293T cells were cotransfected with Luc-EZH2 and wild-type HBP1 or associated mutants. The luciferase activities were expressed as mean ± S.E. from four experiments. *, *p* < 0.05 (*top panel*). Anti-HA immunoblots for HBP1 and mutant protein expression are shown (*center panel*). Also shown is a schematic of wild-type HBP1 and associated mutants (*bottom panel*). *G*, HBP1 inhibits TCF4 binding to its affinity site in the EZH2 promoter *in vitro*. EMSA was performed by using the biotin-labeled probes. Four-microgram amounts of nuclear extracts from HEK293T cells expressing HA-HBP1, HA-pmHMG, or HA-delRepression with or without FLAG-TCF4 were used. The wild-type probe (AGAGTTATATACTG**CTTTGAT**TTGCT) and the mutant probe (AGAGTTATATACTG**CGGGTAT**TTGCT) were used as unlabeled competitors at 100-fold excess. The presence of specific complexes, including supershifted FLAG-TCF4 in the complexes, is indicated (*left panel*). Anti-HA immunoblots for HBP1 and mutant protein expression and anti-FLAG for TCF4 protein expression are shown (*right panel*). *H*, HBP1 binds to the EZH2 promoter through TCF4. H1299 cells were transfected with plasmids indicated in the figure. A ChIP assay was performed with anti-HA antibody or control IgG. The precipitated DNA fragments were amplified with specific oligonucleotides for the EZH2 promoter by real-time PCR. Results are representative of three independent experiments, and values are the mean ± S.E. *, *p* < 0.05. *I*, HBP1 inhibits TCF4 binding to its affinity site in the EZH2 promoter *in vivo*. H1299 cells were transfected with the plasmids indicated in the figure. ChIP assay was performed with anti-FLAG antibody or control IgG. The precipitated DNA fragments were amplified with specific oligonucleotides for the EZH2 promoter by real-time PCR. Results are representative of three independent experiments, and values are the mean ± S.E. *, *p* < 0.05.

To confirm whether HBP1 inhibits Wnt/β-catenin signaling through the LEF/TCF DNA binding site, HEK293T cells were cotransfected with HBP1 and TOPFLASH or FOPFLASH. TOPFLASH contains three LEF/TCF sites followed by the minimal TATA and luciferase. FOPFLASH contains three mutated LEF/TCF sites followed by the minimal TATA and luciferase and is depleted of LEF/TCF binding. 12 h after transfection, cells were treated with LiCl for another 12 h and then harvested for the luciferase assay. As shown in Fig. 7*A*, HBP1 only inhibited the native LEF/TCF element (TOPFLASH) expression activated by LiCl, which is consistent with a previous study (34). In addition, HBP1 could also inhibit Luc-EZH2 transcription activated by SB415286 or LiCl but had no effect on Luc-ΔEZH2 (Fig. 6*C*). Taken together, HBP1 inhibits Wnt/β-catenin signaling through the LEF/TCF DNA binding site.

To determine whether HBP1 inhibits the TCF4 or β-catenin-mediated EZH2 promoter activation, HEK293T cells were cotransfected with HBP1 and Luc-EZH2 and TCF4 or β-catenin and then harvested for the luciferase assay. It turned out that HBP1 could also inhibit the TCF4 or β-catenin-mediated EZH2 transactivation (Fig. 7, *B*, *left panel*, and *C*). However, when the LEF/TCF DNA binding site was deleted, neither TCF4 nor HBP1 affected EZH2 transactivation (Fig. 7*B*, *right panel*). On the other hand, by transfecting HEK293T cells with HBP1, TCF4, or both HBP1 and TCF4, we found that TCF4 rescued EZH2 inhibition by HBP1 (Fig. 7*D*). Furthermore, when β-catenin was knocked down by siRNA, the inhibition of EZH2 expression by HBP1 was enhanced (Fig. 7*E*). To define the functional domains of HBP1 that might be working here, we cotransfected HEK293T cells with Luc-EZH2 and wild-type HBP1 or mutant HBP1, such as mutated HMG box (pmHMG) or deleted repression domain (Delrepression), respectively. As indicated in Fig. 7*F*, wild-type HBP1 and pmHMG both decreased EZH2 promoter activity, whereas the mutant Delrepression hardly affected EZH2 promoter activity. Thus, the repression domain of HBP1 is indispensible for inhibition of the EZH2 promoter by HBP1. These results together suggest that HBP1 inhibits EZH2 expression by blocking Wnt/β-catenin signaling through the repression domain and that the inhibition is not required for DNA binding.

Because HBP1 inhibits TCF4 transcriptional activation by interacting with TCF4 through its repression domain (34), we next asked whether HBP1 inhibits the interaction between TCF4 and the EZH2 promoter in the same way. First, we conducted an EMSA assay to determine the interactions between HBP1, TCF4, and the EZH2 promoter *in vitro*. As shown in Fig. 7*G*, the exogenous TCF4 bound to the EZH2 promoter as expected (*lane 3*), with a supershift by adding anti-FLAG antibody (*lane 4*), which was eliminated by adding either exogenous wild-type HBP1 (*lane 6*) or pmHMG (*lane 9*). Delrepression did not affect the supershift raised by incubation of exogenous TCF4 and anti-FLAG antibody (*lane 11*). These results together suggest that TCF4 binds to the EZH2 promoter and that HBP1 inhibits TCF4 binding to EZH2 through the repression domain.

Then we conducted a ChIP assay to determine the interaction of HBP1, TCF4, and the EZH2 promoter *in vivo*. As shown in Fig. 7*H*, HBP1 or pmHMG alone slightly bound to the EZH2 promoter, whereas Delrepression strongly bound to the EZH2 promoter. However, when TCF4 was knocked down by siRNA, the binding of Delrepression to the EZH2 promoter was drastically decreased. On the other hand, TCF4 alone bound to the EZH2 promoter, and wild-type HBP1 or pmHMG both decreased the binding of TCF4 to the EZH2 promoter, whereas Delrepression did not affect the binding of TCF4 to the EZH2 promoter (Fig. 7*I*). In summary, HBP1 could bind to EZH2 promoter through TCF4, and HBP1 inhibits TCF4 binding to the EZH2 promoter through its repression domain.

As shown in Fig. 6*E*, the decrease of p21 promoter (*left panel*), mRNA, and protein levels (*center* and *right panels*) caused by Wnt/β-catenin signaling activation could be rescued by HBP1 overexpression. These results together suggest that HBP1 inhibits EZH2 transcription through blocking Wnt/β-catenin signaling and finally promotes p21 transcription.

We next asked whether p53 and Wnt signaling regulated by HBP1 are synergistic in induction of p21. As shown in Fig. 5*E*, HBP1 decreased the level of H3K27me3 on the p21 promoter, EZH2 partially rescued the decrease of H3K27me3 on the p21 promoter, and EZH2 overexpression alone increased the H3K27me3 level on the p21 promoter. In contrast to the results, the decrease of H3K27me3 on the p21 promoter facilitated binding the of p53 onto p21 promoter, whereas HBP1-mediated p53 stability enhanced binding (Fig. 2*C*). The data demonstrate that the activation of p53 and repression of Wnt signaling, both by HBP1, are synergistic in induction of p21.

##### HBP1-mediated Activation of p21 through the p53/Mdm2 and TCF4/EZH2 Pathways Contributes to HBP1-induced Premature Senescence and Tumor Inhibition

**FIGURE 8.**
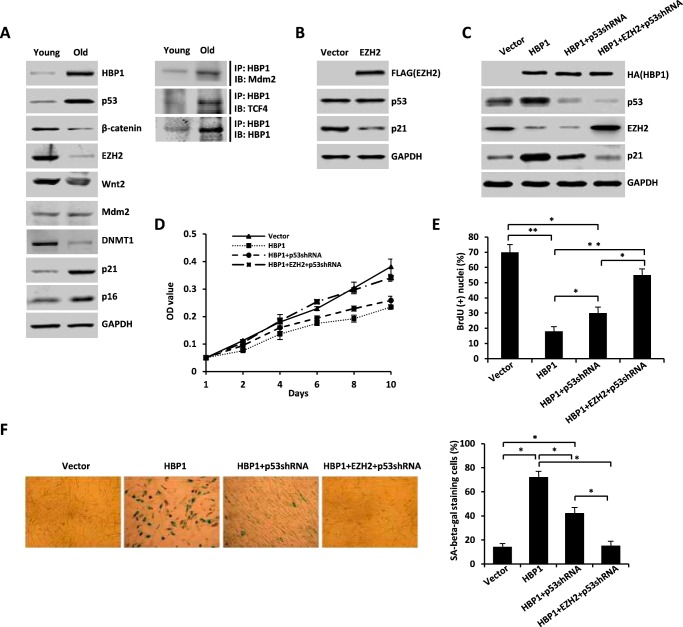

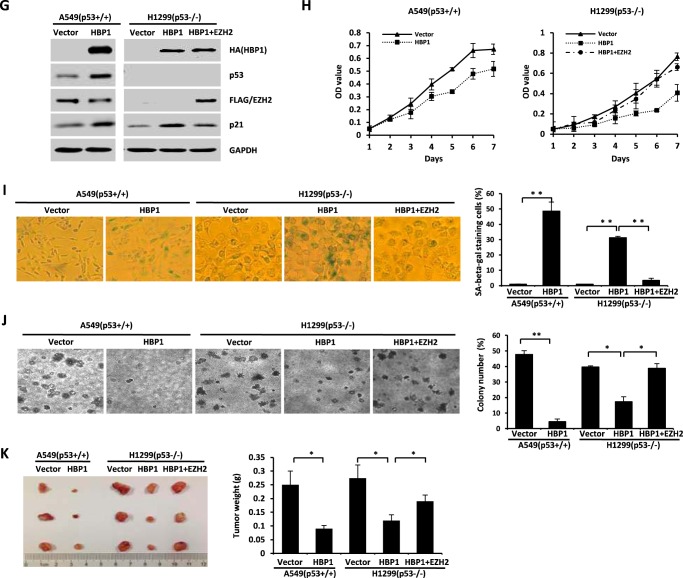
**HBP1-mediated activation of p21 through the Mdm2/p53 and TCF4/EZH2 pathways contributes to HBP1-induced premature senescence and tumor inhibition.** *A*, the protein levels of HBP1, p53, EZH2, and p21 during replicative senescence. Shown is an immunoblot (*IB*) of WI-38 cells in young and old stages. The expression of endogenous HBP1, p53, β-catenin, EZH2, Wnt2, p21, and p16 is shown. GAPDH was used as a loading control (*left panel*). WI-38 cells were lysed with IP lysis buffer and then subjected to immunoprecipitation with anti-HBP1 antibody followed by Western blotting analysis with anti-Mdm2 and anti-TCF4 antibodies (*right panel*). *B*, exogenous EZH2 inhibits p21 expression. The protein levels of EZH2, p53, and p21 were measured by immunoblotting in WI-38 cells (PD20) retrovirally transduced with EZH2 or control vector. *C*, EZH2 attenuates HBP1 elevation of p21 when p53 is knocked down by shRNA. The levels of EZH2, p53, p21, and GAPDH (as a control) were determined by Western blotting of WI-38 cells retrovirally transduced with control vector, HBP1, HBP1 + p53shRNA, or HBP1 + EZH2 + p53shRNA. *D* and *E*, EZH2 attenuates HBP1-induced cell growth arrest when p53 is knocked down by shRNA. MTT (*D*) and BrdU (*E*) incorporation assays were conducted with WI-38 cells retrovirally transduced with control vector, HBP1, HBP1 + p53shRNA, or HBP1 + EZH2 + p53shRNA. The means ± S.E. for three independent experiments are shown. *OD*, optical density. *, *p* < 0.05; **, *p* < 0.01. *F*, EZH2 attenuates HBP1-induced premature senescence when p53 is knocked down by shRNA. WI-38 cells (PD20) were retrovirally transduced with control vector, HBP1, HBP1 + p53shRNA, or HBP1 + EZH2 + p53shRNA. Cells were then stained for SA β-gal on day 14 after transduction (*left panel*). The percentage of cells positive for SA β-gal in WI-38 cells was determined in three independent experiments and expressed as mean ± S.E., with 300 cells/experiment. *, *p* < 0.05 (*right panel*). *G*, EZH2 attenuates HBP1 elevation of p21 in the absence of p53. Levels of EZH2, p53, p21, and GAPDH (as a control) were determined by Western blotting of A549 cells retrovirally transduced with control vector or HBP1 or H1299 cells retrovirally transduced with control vector, HBP1, or HBP1 + EZH2. *H*, EZH2 attenuates HBP1-induced growth inhibition. A549 and H1299 cells were stably transfected with either control vector, HBP1, or HBP1 + EZH2. After puromycin or G418 selection, the growth rates of cells were measured by MTT assay. The mean ± S.E. for three independent experiments are shown. *I*, EZH2 attenuates HBP1-induced premature senescence. A549 and H1299 cells stably transfected with either control vector, HBP1, or HBP1 + EZH2 were subjected to an SA β-gal staining assay (*left panel*). The percentage of cells positive for SA β-gal was determined in three independent experiments and expressed as mean ± S.E., with 300 cells/experiment. **, *p* < 0.01 (*right panel*). *J*, soft agar colony formation assay of A549 and H1299 cells stably transfected with control vector and HBP1 or HBP1 + EZH2. Cells were cultured in soft agar for 2 weeks (*left panel*). The colony numbers in three different microscope fields were counted and are shown as mean ± S.E. *, *p* < 0.05; **, *p* < 0.01 (*right panel*). *K*, A549 and H1299 cells stably transfected with control vector and HBP1 or HBP1 + EZH2 were subcutaneously injected into nude mice. Four weeks after injection, the tumors were weighed, and size was measured. Data are shown as mean ± S.E. (*n* = 3). *, *p* < 0.05.

The above results together suggest that HBP1 could positively regulate the p21 level through both the p53/Mdm2 and TCF4/EZH2 pathways. Finally we explored the biological significance of regulation of p21 by HBP1 through p53/Mdm2 and TCF4/EZH2 signaling. As shown in Fig. 8*A*, *left panel*, HBP1, p53, p21, and p16 protein levels increased during replicative senescence, whereas β-catenin and EZH2, Wnt2, and DNMT1 protein levels decreased during replicative senescence in normal human fibroblast WI-38 cells. We also observed an increase of the endogenous HBP1/Mdm2 interaction and HBP1/TCF4 interaction in senescent cells compared with the young cells (Fig. 8*A*, *right panel*). The data indicate that HBP1-mediated activation of p21 through the p53/Mdm2 and TCF4/EZH2 pathways might contribute to replicative senescence in normal fibroblasts. Overexpression of EZH2 in young WI-38 cells (PD20) resulted in a decrease in p21 protein level but no change in the level of p53 protein (Fig. 8*B*), which indicated that EZH2 decreased p21 independently of p53. As a matter of fact, when p53 was knocked down by shRNA, HBP1 still increased the level of p21 by inhibiting EZH2 (Fig. 8*C*, compare the *third lane* with the *first lane*), which was abolished by additionally overexpressing EZH2 (Fig. 8*C*, compare the *fourth lane* with the *third lane*).

We used a lentivirus to overexpress HBP1, EZH2, and/or p53 shRNA in WI-38 cells. Consistent with our previous studies, HBP1 induced premature senescence, as demonstrated by a decrease of cell growth, assessed by MTT assay (Fig. 8*D*) and BrdU incorporation (Fig. 8*E*), and an increase of SA β-gal staining (Fig. 8*F*). And when p53 was knocked down by shRNA, HBP1 still slightly induced cell growth arrest and premature senescence, whereas overexpression of EZH2 abolished the cell growth arrest and premature senescence induced by HBP1 (Fig. 8, *D–F*). Together, these results suggest that HBP1-mediated activation of p21 through the p53/Mdm2 and TCF4/EZH2 pathways contributes to HBP1-induced premature senescence.

In addition, it has been found that HBP1 can suppress tumorigenesis by inducing cellular senescence (19, 20), so we next investigated whether p21 activation by HBP1 also participates in inhibiting tumorigenesis. As shown in Fig. 8*H*, overexpression of HBP1 inhibited the growth of A549 cells, whereas, in p53-null H1299 cells, HBP1 also caused cell growth arrest. Overexpression of EZH2 rescued the elevation of p21 and the cell growth arrest induced by HBP1 in H1299 cells (Fig. 8, *G* and *H*). Additionally, HBP1 induced cellular senescence both in A549 and H1299 cells, as assessed by SA β-gal staining, and overexpression of EZH2 rescued the cellular senescence induced by HBP1 in H1299 cells (Fig. 8*I*). However, HBP1 inhibited colony formation in soft agar in both A549 and H1299 cells, whereas overexpression of EZH2 rescued colony formation inhibition caused by HBP1 in H1299 cells depleted of p53 (Fig. 8*J*). Finally, HBP1 inhibited tumorigenesis in nude mice inoculated with A549 or H1299 cells, whereas overexpression of EZH2 in H1299 cells rescued tumorigenesis inhibition caused by HBP1 (Fig. 8*K*). Thus, the data further confirm that HBP1-mediated activation of p21 through the p53/Mdm2 and TCF4/EZH2 pathways contributes to HBP1-induced premature senescence and tumor inhibition.

To strengthen a potential clinical significance, we screened and analyzed the differential expression of HBP1, EZH2, and p21 in some human cancers through the TumorProfile database. As shown in supplemental Table 1, although HBP1, EZH2, and p21 were all up-regulated in some brain cancers, such as astrocytoma, ependymoma, and glioblastoma, HBP1 was significantly down-regulated in more cancers compared with their normal or non-cancerous tissues at the mRNA level, such as breast cancer, colorectal adenocarcinoma, hepatocellular carcinoma, lung adenocarcinoma, lung squamous cell carcinoma, and pancreatic cancer, whereas EZH2 was significantly up-regulated in these cancers, further confirming the negative correlation between HBP1 and EZH2. Similar to HBP1, p21 was down-regulated in colorectal adenocarcinoma, lung adenocarcinoma, and lung squamous cell carcinoma, and so forth (supplemental Table 1, Fig. 1*A*). The BioXpress database was constructed by using the dataset deposited in the Cancer Genome Atlas (41). Based on RNA sequencing from the database, HBP1 and p21 were frequently down-regulated in uterine cancer, colon cancer, rectum cancer, lung cancer, liver cancer, and stomach cancer, whereas EZH2 was frequently up-regulated in these cancers (supplemental Fig. S2). The results from either array or RNA sequencing data supported the negative correlation between HBP1 and EZH2, whereas positive correlation between HBP1 and p21 also existed in cancers and suggested their potential roles in regulation of tumorigenesis for many cancer types.

## Discussion

P21, as a CDK inhibitor, plays an important role in cell cycle arrest. Changes in p21 expression are also associated with aging, cellular senescence, and tumorigenesis. Therefore, proper regulation of p21 levels and activity is critical for maintaining cells in a differentiated state and preventing tumorigenesis. Here we identify HBP1 as an intriguing and important factor in regulating the p21 state in senescence and tumorigenesis.

### 

#### 

##### HBP1 Is an Important Transcription Factor That Regulates p21 Levels through Multiple Mechanisms

**FIGURE 9.**
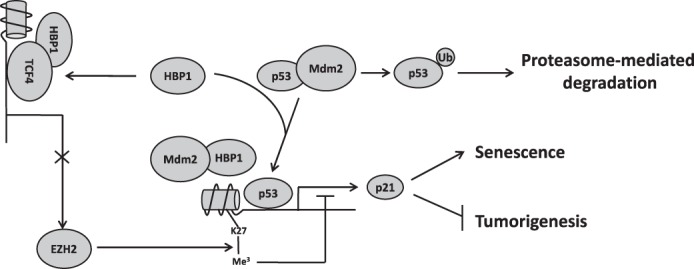
**Model for the regulation of p21 by HBP1 and its role in senescence induction and tumor inhibition.** HBP1 can positively regulate p21 expression through both the p53/Mdm2 pathway and TCF4/EZH2 pathway. HBP1 binds Mdm2 through the repression domain and inhibits the interaction between p53 and Mdm2, thus abrogating Mdm2-mediated p53 degradation. The stabilized p53 then activates p21 transcription and leads to p21 elevation. HBP1 can also bind to TCF4 and inhibit the activation of EZH2 transcription by TCF4, resulting in a decrease in the level of H3K27me3 on the p21 promoter. The H3K27me3 hypomethylation state of the p21 promoter enhances p53 binding and p21 transcriptional activation. Overall, HBP1-mediated activation of p21 through Mdm2/p53 and TCF4/EZH2 pathways synergistically contributes to HBP1-induced premature senescence and tumor inhibition.

The data in this paper support three conclusions that are represented in the model in Fig. 9. First, HBP1 elevates p21 levels by enhancing p53 protein stability, which is a positive regulator of p21. We identified that HBP1 enhances p53 protein stability through reducing ubiquitination-proteasomal degradation by Mdm2 (Figs. 1–4). Second, EZH2 is a novel target of Wnt/β-catenin signaling. β-catenin/TCF4 directly activates EZH2 transcription. Third, HBP1 also elevates p21 levels through inhibiting EZH2 transcription by Wnt/β-catenin signaling, thus decreasing the H3K27me3 level of overall and specific histone on the p21 promoter, resulting in p21 transactivation. It was reported that HBP1 is an inhibitor of Wnt/β-catenin signaling (34). We document here that HBP1 inhibits EZH2 expression by repressing TCF4 binding to the EZH2 promoter. Together, our work is consistent with a model in which HBP1 up-regulates p21 by both enhancing p53 stability and inhibiting EZH2 expression. The multiple mechanisms are involved in HBP1-mediated senescence induction and tumor inhibition.

The p21 CDK inhibitor is a critical factor in the inhibition of G_1_ progression by blocking the activity of CDKs. In separate transgenic mouse models, both HBP1 and p21 blocked G_1_ progression in liver regeneration (42, 43). Intriguingly, a previous paper reported that HBP1 could repress the p21 promoter in Caco-2 cells, which were derived from colon adenocarcinoma (44). The investigators performed a chloramphenicol acetyltransferase (CAT) assay to demonstrate that HBP1 could repress basal and E2F-induced activity of the p21 promoter, thereby antagonizing an E2F-induced increase of p21 in Caco-2 cells during differentiation. The report is in contrast to our results. Our previous papers identified that HBP1 is a dual transcription factor. It can act either as a repressor or as an activator, depending on the acetylation state and promoter sequences (19). From the repressor standpoint, the DNMT1, p47phox (18), and N-myc (45) genes are genes that are repressed by HBP1 in a sequence-specific manner. From the activator standpoint, p16 (20), p21 (this work), myeloperoxidase (46), and histone H1 (47) genes are genes that are transcriptionally activated by HBP1. Therefore, we suppose that the complex transcriptional regulation of p21 in different cell lines is perhaps due to HBP1 differential acetylation or promoter binding site in different physical status. Therefore, more studies are needed to clarify the mechanism. Actually, lines of evidence indicate that HBP1 mainly functions as an inhibitor of cell growth. For instance, HBP1 could delay G_1_ progression by increasing the p21 level in liver regeneration and cell differentiation (44, 48). HBP1 also induces cell growth arrest by up-regulating p16 and inhibiting DNMT1 expression (19, 20). Hence, we suppose that the positive regulation of p21 by HBP1 might overtake the inhibition of p21 by HBP1 when HBP1 functions mainly as an inhibitor of cell growth; thus, the total effect of HBP1 on p21 would be elevating p21 levels. As indicated in our study, HBP1 increases p21 protein levels through opposite abilities of the p53/p21 pathway and Wnt/β-catenin/EZH2 signaling. On one hand, HBP1 interacts with Mdm2 E3 ubiquitin ligase to inhibit p53 ubiquitinal degradation, thus augmenting p53/p21 signaling by enhancing p53 stability. On the other hand, HBP1 elevates p21 transcription through inhibiting Wnt/β-catenin/TCF4/EZH2 signaling. EZH2 is identified as a novel target of Wnt/β-catenin signaling. HBP1 decreases TCF4-mediated transactivating EZH2, thus resulting in overall H3K27me3 hypomethylation, especially in the p21 promoter. The histone hypomethylation state in the promoter encourages p21 expression. Additionally, our previous data indicated that HBP1 could induce DNA hypomethylation in the p21 promoter by inhibiting DNA methyltransferse 1 (DNMT1) expression (19). Based on our current and previous data, we conclude that HBP1 up-regulates p21 levels either by maintaining histone and DNA hypomethylation in the promoter or by enhancing p21 transcription by p53.

##### Relevance to Senescence and Cancer

Together, the data in this article indicate that HBP1 has a central role in regulating p21 and other genes. This study gives important insights into how the reduction of a single factor can abrogate premature senescence to begin a cascade of events that lead to full tumorigenesis. The complex mechanisms of HBP1-mediated repression, activation, and histone methylation are all required to orchestrate senescence and prevent tumorigenesis. The likely consequence is that the HBP1-mediated repression of EZH2 and DNMT1 triggers the global hypomethylation of histone and DNA. When we investigated the p21 gene, we found that the H3K27me3 hypomethylation state of the p21 promoter enhanced p53 binding and transcriptional activation. Additionally, HBP1-mediated p53 stability also enhanced the transactivation. Thus, both functions of HBP1 were synergistic and required for enacting senescence and tumor inhibition. Our work underscores the importance of the HBP1-EZH2/p53-p21 axis for regulating cellular senescence because its abrogation disrupts senescence and promotes tumorigenesis.

In the context of cancer, several lines of evidence underscore the importance of the HBP1 transcription factor. We had reported previously that HBP1 was decreased in invasive breast cancer and correlated with a poor prognosis (49). Other reports suggest a similar role for a decrease in HBP1 in prostate cancer (50). Sears and co-workers (51) also showed that HBP1 inhibits the activity of Myc, a gene often overexpressed in breast cancer. Our own inspection of the public databases further added the observation that reduced HBP1 and p21 levels were statistically associated with elevated EZH2 levels (Fig. 1*A* and supplemental Fig. S2 and Table 1). The variation in the expression levels of HBP1, p21, and EZH2, which are all associated with premature senescence, could have a lasting impact on tumorigenic progression. For HBP1, we would suggest that decreased HBP1 expression might contribute to interruption of senescence and the deregulation of some signaling pathways (Wnt, EGF receptor) that are linked to a poor prognosis in breast and other cancers. Although only a first glimpse, the bioinformatics analysis underscores the potential future clinical relevance of the studies in this paper. More studies are needed to investigate the possible prognostic and therapeutic potential of HBP1, p21, and EZH2 in cancers.

## Author Contributions

Y. C. performed and analyzed the experiments and contributed to manuscript writing. K. P. performed the protein half-life assay and coordinated the study. P. W. performed the bioinformatics analysis. Z. C. performed the real-time PCR assay and contributed to the bioinformatics analysis. W. W. wrote the paper. S. W., N. H., and J. X. provided technical assistance and contributed to the preparation of the figures. H. L. and W. J. contributed to the GST pulldown assay. G. L. contributed to manuscript writing. X. Z. coordinated the study and wrote the paper. All authors reviewed the results and approved the final version of the manuscript.

## Supplementary Material

Supplemental Data

## Footnotes

3 Y. Chen, K. Pan, P. Wang, Z. Cao, W. Wang, S. Wang, N. Hu, J. Xue, H. Li, W. Jiang, G. Li, and X. Zhang, unpublished data.

## Footnotes

2 The abbreviations used are:

CDK: cyclin-dependent kinase
HMG: high-mobility group
H3K27me3: trimethylation of histone H3 at lysine 27
LEF: lymphoid enhancer factor-1
TCF: T-cell factor
IP: immunoprecipitation
MTT: 3-(4,5-dimethylthyazol-2-yl)-2,5-diphenyltetrazolium bromide
SA: senescence-associated.
