# Supplementary material for: HBP1-mediated Regulation of p21 Protein through the Mdm2/p53 and TCF4/EZH2 Pathways and Its Impact on Cell Senescence and Tumorigenesis
Source: J Biol Chem. 2016 Apr 21;291(24):12688–705. doi: 10.1074/jbc.M116.714147 (PMC4933444; doi:10.1074/jbc.M116.714147)
Supplement: Supplemental Data [file supp_291_24_12688__index.html]

HBP1-mediated regulation of p21 through Mdm2/p53 and TCF4/EZH2 pathways and its impact on cell senescence and tumorigenesis — HBP1-mediated Regulation of p21 Protein through the Mdm2/p53 and TCF4/EZH2 Pathways and Its Impact on Cell Senescence and Tumorigenesis — HBP1 Is a Positive Regulator of p21 — Supplemental Data 

# HBP1-mediated Regulation of p21 Protein through the Mdm2/p53 and TCF4/EZH2 Pathways and Its Impact on Cell Senescence and Tumorigenesis

## Supplemental Data

- Supplementary Figure Legends and figures (.pdf, 274 KB) - Supplementary Figure Legends and figures
- Supplementary Table 1 (.pdf, 121 KB) - Supplementary Table 1
