# Supplementary material for: HBP1-mediated Regulation of p21 Protein through the Mdm2/p53 and TCF4/EZH2 Pathways and Its Impact on Cell Senescence and Tumorigenesis
Source: J Biol Chem. 2016 Apr 21;291(24):12688–705. doi: 10.1074/jbc.M116.714147 (PMC4933444; doi:10.1074/jbc.M116.714147)
Supplement: Supplemental Data [file 10.1074_M116.714147_jbc.M116.714147-1.pdf]

SUPPLEMENTARY FIGURE LEGENDS

FIGURE S1. Knockdown of EZH2 rescues the phenotype in HBP1 depleted cells. (A) MTT assay was conducted with H1299 cells stably transfected with pLL3.7, pLL3.7-shHBP1, pLL3.7-shEZH2 or pLL3.7-shHBP1 + pLL3.7-shEZH2. The mean ±S.E. for three independent experiments are shown. OD, optical density. (B) Soft agar colony formation assay was conducted with H1299 cells stably transfected with pLL3.7, pLL3.7-shHBP1, pLL3.7-shEZH2 or pLL3.7-shHBP1 + pLL3.7-shEZH2. Cells were cultured in soft agar for 2 weeks (top). The colony numbers in three different microscope fields were counted and are shown as mean ±S.E. *, $p<0.05$, **, $p<0.01$. (bottom).

FIGURE S2. The retrieval results from the BioXpress database web server. The x-axis indicates cancer type, while the y-axis indicates the dysregulated frequency of the queried gene in the corresponding cancer samples. We downloaded the well-processed data set from the the BioXpress database, which was constructed by using the data set from TCGA, and analyzed the expression of HBP1(A), EZH2(B) and p21(C). Abbreviations for cancer type are shown below: Esophageal cancer [EC]; Cervical Cancer [Cerca]; Uterine cancer [Uteca]; Liver Cancer [Livca]; Kidney cancer [Kidca]; Colon Cancer [Colca]; Rectum cancer [Recca]; Pancreatic Cancer [PACA]; Thyroid cancer [Thyca]; Breast Cancer [BRCA]; Lung Cancer [Lunca]; Head and neck cancer [H&NC]; Urinary bladder cancer [UBC]; Stomach cancer [Stoca]; Prostate cancer [PCa].

A

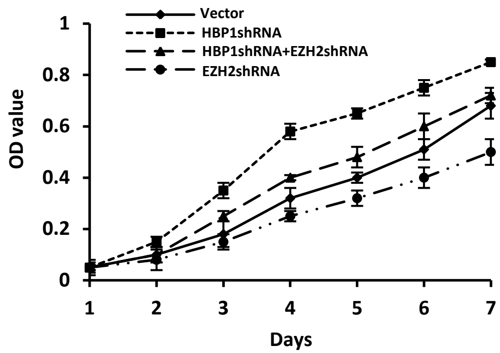
Vector
HBP1shRNA
HBP1shRNA+EZH2shRNA
EZH2shRNA
OD value
Days

B

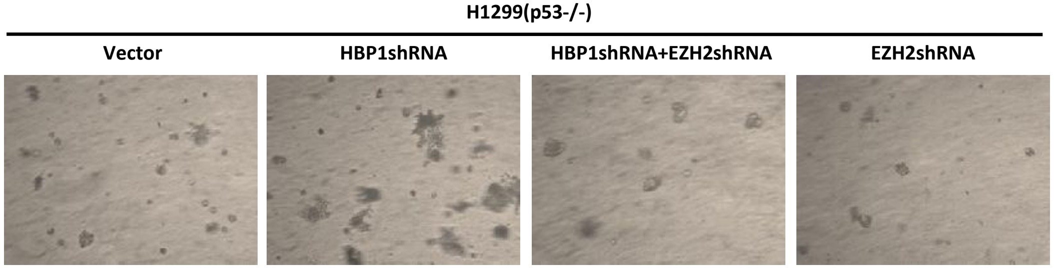
H1299(p53-/-)
Vector
HBP1shRNA
HBP1shRNA+EZH2shRNA
EZH2shRNA

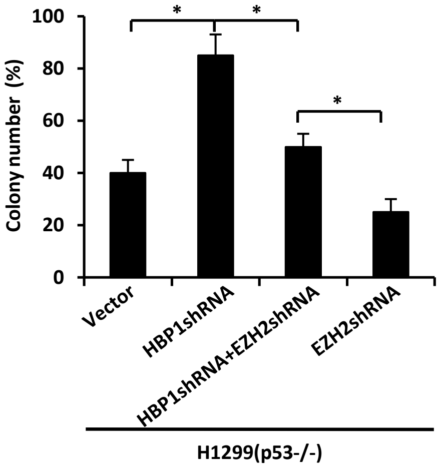
Colony number (%)
Vector
HBP1shRNA
HBP1shRNA+EZH2shRNA
EZH2shRNA
H1299(p53-/-)

Figure S1

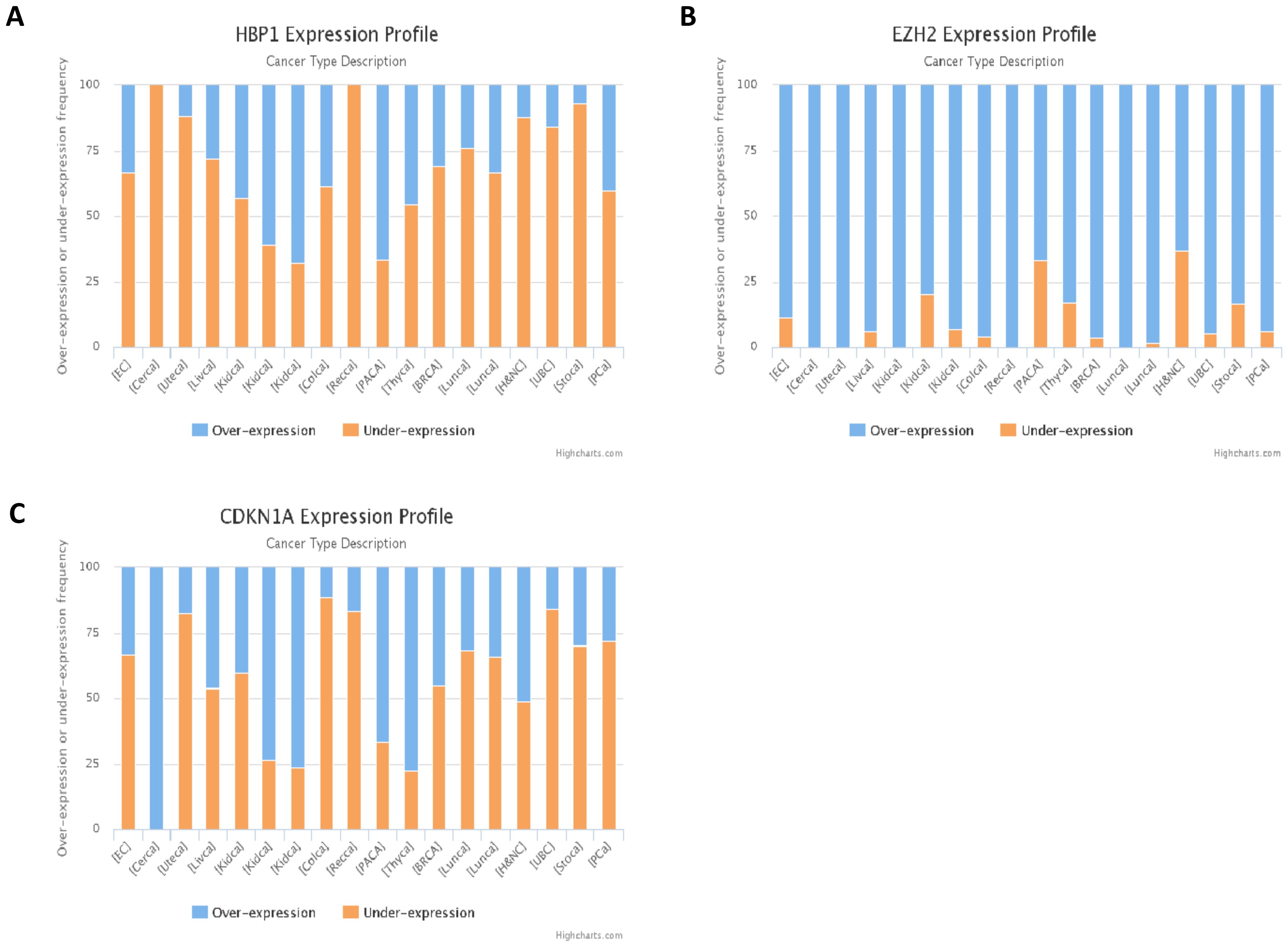
A
HBP1 Expression Profile
Cancer Type Description
Over-expression or under-expression frequency
100
75
50
25
0
[EC]
[Cercal]
[Utecal]
[Livcal]
[Kidcal]
[Kidcal]
[Kidcal]
[Colcal]
[Reccal]
[PACA]
[Thycal]
[BRCA]
[Luncal]
[Luncal]
[H&NC]
[UBC]
[Stocal]
[PCal]
Over-expression
Under-expression
Highcharts.com
B
EZH2 Expression Profile
Cancer Type Description
C
CDKN1A Expression Profile
Cancer Type Description

Figure S2
