# Supplementary material for: HBP1-mediated Regulation of p21 Protein through the Mdm2/p53 and TCF4/EZH2 Pathways and Its Impact on Cell Senescence and Tumorigenesis
Source: J Biol Chem. 2016 Apr 21;291(24):12688–705. doi: 10.1074/jbc.M116.714147 (PMC4933444; doi:10.1074/jbc.M116.714147)
Supplement: Supplemental Data [file 10.1074_M116.714147_jbc.M116.714147-2.pdf]

SUPPLEMENTARY TABLE 1. The average expression intensities of HBP1, EZH2 and p21 in multiple cancers.

Tissue	Sample size	HBP1			EZH2			p21		
		ARS[a]	*P*-value[b]	Bonferroni[c]	ARS	*P*-value	Bonferroni	ARS	*P*-value	Bonferroni
bladder cancer	186	85.57	0.8944376	1	74.83	4.33E-15	2.36E-10	87.76	5.10E-05	1
bladder control	64	76.14			52.55			75.95		
astrocytoma	207	90.82	3.65E-22	1.99E-17	65.54	1.09E-30	5.97E-26	88.66	1.58E-21	8.64E-17
ependymoma	152	89.84	4.94E-12	2.70E-07	71.84	7.64E-37	4.17E-32	90.51	6.15E-27	3.36E-22
glioblastoma	498	90.54	7.49E-22	4.09E-17	82.77	4.46E-54	2.44E-49	87.69	4.70E-22	2.57E-17
medulloblastoma	302	92.06	1.49E-30	8.13E-26	92.29	2.77E-52	1.51E-47	72.77	0.00050112	1
meningioma	89	92.28	3.05E-24	1.67E-19	61.09	1.48E-19	8.10E-15	91.98	3.79E-23	2.07E-18
neuroblastoma	221	91.50	2.40E-24	1.31E-19	85.85	1.08E-45	5.87E-41	83.18	5.18E-07	0.028243796
oligodendroglioma	117	88.67	7.29E-06	0.397805619	75.32	6.26E-28	3.42E-23	84.37	1.40E-08	0.000765794
retinoblastoma	78	82.14	0.0154583	1	97.47	5.17E-31	2.82E-26	89.74	1.09E-20	5.96E-16
brain control	104	85.88			35.90			75.75		
breast cancer	3080	93.65	9.17E-25	5.01E-20	78.43	7.47E-130	4.08E-125	87.27	0.32876776	1
breast control	374	94.56			56.31			87.55		
colorectal adenocarcinoma	1841	91.46	7.52E-34	4.10E-29	84.65	3.45E-86	1.88E-81	91.12	3.44E-59	1.87E-54
colon mucosa normal	273	93.30			75.62			95.38		
gastric cancer	681	92.33	0.0526557	1	79.15	6.23E-12	3.39E-07	90.87	0.00579905	1
gaster control	61	92.02			69.18			91.33		
head and neck squamous cell carcinoma	220	91.64	3.86E-05	1	88.79	9.22E-09	0.0005035	92.55	0.80009657	1
head and neck squamous cell control	59	93.53			82.51			92.78		
ccRCC	652	94.17	3.13E-06	0.170801273	66.93	7.09E-85	3.87E-80	93.13	1.30E-46	7.12E-42
kidney control	244	93.77			40.37			85.53		
hepatocellular carcinoma	263	92.45	0.0001599	1	77.44	2.66E-29	1.45E-24	87.27	0.53621843	1
liver control	62	94.50			51.18			87.85		
lung adenocarcinoma	1158	91.62	8.55E-39	4.67E-34	75.85	7.98E-111	4.36E-106	89.47	4.81E-53	2.62E-48
lung squamous cell carcinoma	422	90.09	8.59E-41	4.69E-36	82.05	6.44E-97	3.52E-92	91.16	1.19E-21	6.48E-17
lung control	333	94.37			58.63			94.31		
oral squamous cell carcinoma	339	93.57	2.21E-09	0.00012053	81.35	0.39199038	1	94.84	0.00059649	1
oral squamous cell control	118	94.14			81.50			92.90		
ovarian cancer	379	90.89	0.0016606	1	80.54	3.40E-22	1.86E-17	84.98	0.00190026	1
ovary control	120	92.76			69.79			88.83		
pancreatic cancer	263	89.47	3.73E-11	2.04E-06	74.64	4.73E-21	2.58E-16	92.59	0.66870137	1

pancreas control	83	94.14			59.69			91.96		
prostate cancer	345	94.30	9.93E-08	0.005422575	64.75	9.51E-05	1	88.81	1.20E-05	0.655455373
prostate control	81	90.98			53.47			91.60		
skin melanoma	522	93.25	1.73E-36	9.44E-32	81.20	0.00014746	1	88.98	1.40E-12	7.63E-08
skin normal	305	95.44			79.69			93.25		

a: ARS denotes the average rank score.
b: The *P*-values were calculated using the Wilcoxon rank-sum test in R (http://www.r-project.org/) software environment as compared to the corresponding normal or non-tumour tissues.
c: The P-values were adjusted using the Bonferroni correction method by the function "p.adjust" in R software. Bonferroni corrected p values ≤ 0.05 were considered as statistically significant differences.
